# (*C-meso-N-meso*-5,12-Dimethyl-7,14-diphenyl-1,4,8,11-tetra­aza­cyclo­tetra­deca-4,11-diene)copper(II) bis­[*O*,*O*′-bis­(4-methyl­phen­yl)dithio­phosphate]

**DOI:** 10.1107/S1600536810046672

**Published:** 2010-11-17

**Authors:** Li-Ke Zou, Bin Xie, Jian-Shen Feng, Chuan Lai

**Affiliations:** a College of Chemistry and Pharmaceutical Engineering, Sichuan University of Science and Engineering, 643000 Zigong, Sichuan, People’s Republic of China

## Abstract

In the title compound, [Cu(C_24_H_32_N_4_)](C_14_H_14_O_2_PS_2_)_2_, the Cu^II^ atom lies on an inversion center and is chelated by the macrocyclic ligand in a distorted CuN_4_ square-planar geometry. Two *O*,*O*′-bis­(4-methyl­phen­yl)dithio­phosphate anions occupy the axial positions with long Cu⋯S distances of 3.0090 (8) Å. Inter­molecular N—H⋯S and C—H⋯S hydrogen bonding is present between the anions and the cation.

## Related literature

For bond-length data, see: Allen *et al.* (1987 ▶). For complexes of Cu^I^ and Cu^II^ with *O*,*O*′-dialkyl­dithio­phosphate (DPP) ligands, see: Drew *et al.* (1987 ▶); Liaw *et al.* (2005 ▶). For the ability of Cu^II^ to form high nuclearity clusters, see: Liu *et al.* (1995 ▶); Li *et al.* (2008 ▶). For related structures, see: Feng *et al.* (2009 ▶); Xie *et al.* (2009 ▶); He *et al.* (2010 ▶). For the synthesis, see: Curtis (2001 ▶).
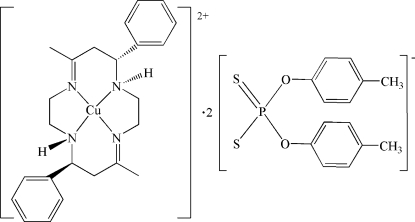


## Experimental

### 

#### Crystal data

[Cu(C_24_H_32_N_4_)](C_14_H_14_O_2_PS_2_)_2_
*M* *_r_* = 1058.76
Monoclinic, 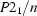

*a* = 9.9467 (18) Å
*b* = 19.829 (3) Å
*c* = 13.550 (2) Å
β = 107.563 (2)°
*V* = 2548.0 (8) Å^3^
*Z* = 2
Mo *K*α radiation
μ = 0.70 mm^−1^
*T* = 103 K
0.43 × 0.27 × 0.17 mm

#### Data collection

Rigaku SPIDER diffractometer
Absorption correction: multi-scan (*ABSCOR*; Higashi, 1995 ▶) *T*_min_ = 0.750, *T*_max_ = 0.890
19915 measured reflections
5836 independent reflections
4826 reflections with *I* > 2σ(*I*)
*R*_int_ = 0.037

#### Refinement

*R*[*F* ^2^ > 2σ(*F* ^2^)] = 0.039
*wR*(*F* ^2^) = 0.093
*S* = 1.00
5836 reflections
311 parameters
H atoms treated by a mixture of independent and constrained refinement
Δρ_max_ = 0.39 e Å^−3^
Δρ_min_ = −0.29 e Å^−3^

### 

Data collection: *RAPID-AUTO* (Rigaku, 2004 ▶); cell refinement: *RAPID-AUTO*; data reduction: *RAPID-AUTO*; program(s) used to solve structure: *SHELXS97* (Sheldrick, 2008 ▶); program(s) used to refine structure: *SHELXS97* (Sheldrick, 2008 ▶); molecular graphics: *ORTEP-3 for Windows* (Farrugia, 1997 ▶); software used to prepare material for publication: *SHELXL97*.

## Supplementary Material

Crystal structure: contains datablocks I, global. DOI: 10.1107/S1600536810046672/xu5079sup1.cif

Structure factors: contains datablocks I. DOI: 10.1107/S1600536810046672/xu5079Isup2.hkl

Additional supplementary materials:  crystallographic information; 3D view; checkCIF report

## Figures and Tables

**Table 1:** Hydrogen-bond geometry (Å, °)

*D*—H⋯*A*	*D*—H	H⋯*A*	*D*⋯*A*	*D*—H⋯*A*
N1—H1*N*⋯S2^i^	0.81 (2)	2.73 (2)	3.4868 (19)	157
C6—H6*C*⋯S2^ii^	0.98	2.80	3.755 (2)	164

Symmetry codes: (i) 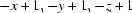; (ii) 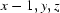.

**Fig. 1.**
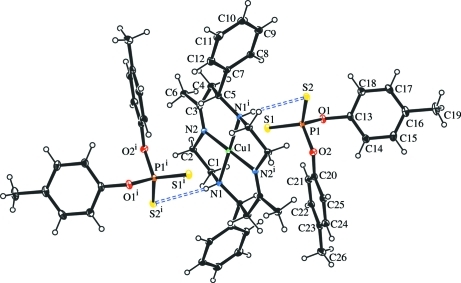
The molecular structure of compound, showing the atom-numbering scheme. Displacement ellipsoids are drawn at the 30% probability level. H atoms are represented as small spheres of arbitrary radii. Hydrogen-bonds are shown as dashed lines [symmetry code: (i) -x + 1, -y + 1, -z + 1].

## supplementary crystallographic information

### Comment

The complexes of Cu^I^ and Cu^II^ with *O*,*O*' -dialkyldithiophosphate ligands (DDP), have been explored extensively in the past decades because of their potential use as anti-oxidants, additives to lubricating oils, flotation reagents, insecticides (Drew *et al.*, 1987; Liaw *et al.*, 2005). A remarkable feature of the Cu^I^ is its ability to form high nuclearity clusters, in which the DDP ligands possess a variety of bridge bonding characteristics (Liu *et al.*, 1995; Li *et al.*, 2008). However, the reactions between Cu^II^ and DDP rarely give stable Cu^II^ complexes because the Cu^II^ atom is readily reduced by DDP to form Cu^I^ clusters. When reacting with DDP, the Cu^II^ can be stabilized by the formation of adducts with tetradentate nitrogen-donor ligands, *e.g.* macrocyclic tetramine. We have recently reported several structures of such kind of adducts (Feng *et al.*, 2009; He *et al.*, 2010). Herein, we report the structure of [Cu(*meso*-diphenyl[14]dien)] [S_2_P(OC_6_H_4_Me-4)_2_]_2_, where *meso*-diphenyl[14]dien is *C-meso-N-meso*-5,12-dimethyl-7,14- diphenyl-1,4,8,11-tetraazacyclotetradeca-4,11-diene.

The molecular of the title adduct comprises a complex cation [Cu(*meso*-diphenyl[14]dien)]^2+^ and two uncoordinated [S_2_P(OC_6_H_4_Me-4)_2_]^-^ anion. Its structure is remarkably similar to the analogues, [Cu(*trans*-[14]dien)][S_2_P(OC_6_H_4_Me-4)_2_]_2_ (He *et al.*, 2010) and ([Ni(*trans*-[14]dien)] [S_2_P(OC_6_H_4_Me-4)_2_]_2_ (Xie *et al.*, 2009), where *trans*-[14]dien is *meso*-5,7,7,12,14,14-hexamethyl-1, 4,8,11-tetraazacyclotetradeca-4,11-diene. The Cu^II^ atom, lying on an inversion centre, is coordinated by four N atoms from the macrocyclic tetramine *meso*-diphenyl[14]dien and adopts a relatively undistorted square-planar geometry (Fig.1). The two [S_2_P(OC_6_H_4_Me-4)_2_]^-^ anion, occupying the axial positions to form an octahedral asymmetric unit, only act as counter-ions to balance the charge and interact with the complex cation through N—H···S hydrogen bonds (Table 1). All the bond lengths and angles in the complex are generally within normal ranges (Allen *et al.*, 1987).

### Experimental

5,12-Dimethyl-7,14-diphenyl-1,4,8,11-tetraazacyclotetradeca-4,11-diene (diphenyl[14]dien) was synthesized according to the procedure described by Curtis (2001).

A hot solution of  CuCl_2_.H_2_O (1 mmol, 0.171 g) and diphenyl[14]dien (1 mmol, 0.376 g) in 20 ml ethanol was quickly added to [Et_2_NH_2_][S_2_P(OC_6_H_4_Me-4)_2_] (2 mmol, 0.823 g) dissolved in 20 ml hot ethanol with stirring. The mixture was refluxed for 4 h and refrigerated overnight, the purple product was filtered off, washed successively with water, methanol, ether and then air dried. The crude product was dissolved in hot dimethylformamide and filtered. The filtrate was kept at room temperature and violet block crystals suitable for X-ray diffraction studies were obtained after two months.

### Refinement

H atoms on C were fixed geometrically and treated as riding, with C—H = 1.00 (methine), 0.99 (methylene), 0.98 (methyl) or 0.95 Å (aromatic) and *U*_iso_(H) = 1.2*U*_eq_(C). H atom on N was located in a difference Fourier map and refined isotropically.

### Crystal data

**Table:**

[Cu(C_24_H_32_N_4_)](C_14_H_14_O_2_PS_2_)_2_	*F*(000) = 1110
*M**_r_* = 1058.76	*D*_x_ = 1.380 Mg m^−^^3^
Monoclinic, *P*2_1_/*n*	Mo *K*α radiation, λ = 0.71073 Å
Hall symbol: -P 2yn	Cell parameters from 6736 reflections
*a* = 9.9467 (18) Å	θ = 3.0–27.5°
*b* = 19.829 (3) Å	µ = 0.70 mm^−^^1^
*c* = 13.550 (2) Å	*T* = 103 K
β = 107.563 (2)°	Block, violet
*V* = 2548.0 (8) Å^3^	0.43 × 0.27 × 0.17 mm
*Z* = 2	

### Data collection

**Table:**

Rigaku SPIDER diffractometer	5836 independent reflections
Radiation source: Rotating Anode	4826 reflections with *I* > 2σ(*I*)
graphite	*R*_int_ = 0.037
ω scans	θ_max_ = 27.5°, θ_min_ = 3.1°
Absorption correction: multi-scan (*ABSCOR*; Higashi, 1995)	*h* = −12→12
*T*_min_ = 0.750, *T*_max_ = 0.890	*k* = −18→25
19915 measured reflections	*l* = −15→17

### Refinement

**Table:**

Refinement on *F*^2^	Primary atom site location: structure-invariant direct methods
Least-squares matrix: full	Secondary atom site location: difference Fourier map
*R*[*F*^2^ > 2σ(*F*^2^)] = 0.039	Hydrogen site location: inferred from neighbouring sites
*wR*(*F*^2^) = 0.093	H atoms treated by a mixture of independent and constrained refinement
*S* = 1.00	*w* = 1/[σ^2^(*F*_o_^2^) + (0.0451*P*)^2^ + 1.360*P*] where *P* = (*F*_o_^2^ + 2*F*_c_^2^)/3
5836 reflections	(Δ/σ)_max_ = 0.001
311 parameters	Δρ_max_ = 0.39 e Å^−^^3^
0 restraints	Δρ_min_ = −0.29 e Å^−^^3^

### Special details

**Table:**

Geometry. All e.s.d.'s (except the e.s.d. in the dihedral angle between two l.s. planes) are estimated using the full covariance matrix. The cell e.s.d.'s are taken into account individually in the estimation of e.s.d.'s in distances, angles and torsion angles; correlations between e.s.d.'s in cell parameters are only used when they are defined by crystal symmetry. An approximate (isotropic) treatment of cell e.s.d.'s is used for estimating e.s.d.'s involving l.s. planes.
Refinement. Refinement of *F*^2^ against ALL reflections. The weighted *R*-factor *wR* and goodness of fit *S* are based on *F*^2^, conventional *R*-factors *R* are based on *F*, with *F* set to zero for negative *F*^2^. The threshold expression of *F*^2^ > σ(*F*^2^) is used only for calculating *R*-factors(gt) *etc*. and is not relevant to the choice of reflections for refinement. *R*-factors based on *F*^2^ are statistically about twice as large as those based on *F*, and *R*- factors based on ALL data will be even larger.

### Fractional atomic coordinates and isotropic or equivalent isotropic displacement parameters (Å^2^)

**Table:**

	*x*	*y*	*z*	*U*_iso_*/*U*_eq_	
Cu1	0.5000	0.5000	0.5000	0.01698 (10)	
P1	0.73029 (5)	0.58172 (3)	0.33214 (4)	0.01667 (12)	
S1	0.53338 (5)	0.59003 (3)	0.33017 (4)	0.02142 (12)	
S2	0.87798 (5)	0.61491 (3)	0.45230 (4)	0.02187 (12)	
O1	0.73913 (15)	0.61604 (7)	0.22511 (11)	0.0209 (3)	
O2	0.77276 (15)	0.50382 (7)	0.31829 (11)	0.0196 (3)	
N1	0.34219 (17)	0.44424 (8)	0.40943 (13)	0.0142 (3)	
N2	0.35057 (17)	0.56801 (8)	0.49380 (13)	0.0153 (3)	
C1	0.2328 (2)	0.49252 (10)	0.35271 (16)	0.0175 (4)	
H1A	0.1433	0.4684	0.3200	0.021*	
H1B	0.2628	0.5150	0.2976	0.021*	
C2	0.2112 (2)	0.54459 (10)	0.42844 (16)	0.0181 (4)	
H2A	0.1562	0.5831	0.3903	0.022*	
H2B	0.1581	0.5244	0.4723	0.022*	
C3	0.3657 (2)	0.62765 (10)	0.53057 (16)	0.0168 (4)	
C4	0.5066 (2)	0.65355 (10)	0.59550 (18)	0.0215 (4)	
H4A	0.4890	0.6873	0.6440	0.026*	
H4B	0.5497	0.6779	0.5489	0.026*	
C5	0.6161 (2)	0.60396 (10)	0.65898 (15)	0.0166 (4)	
H5	0.5719	0.5778	0.7042	0.020*	
C6	0.2489 (2)	0.67873 (10)	0.50813 (17)	0.0212 (4)	
H6A	0.2389	0.7000	0.4410	0.025*	
H6B	0.2712	0.7133	0.5624	0.025*	
H6C	0.1604	0.6563	0.5064	0.025*	
C7	0.7405 (2)	0.64289 (10)	0.72869 (15)	0.0172 (4)	
C8	0.8229 (2)	0.68434 (10)	0.68628 (16)	0.0196 (4)	
H8	0.8024	0.6874	0.6133	0.024*	
C9	0.9344 (2)	0.72098 (11)	0.75002 (17)	0.0235 (5)	
H9	0.9897	0.7489	0.7205	0.028*	
C10	0.9650 (2)	0.71691 (11)	0.85647 (18)	0.0274 (5)	
H10	1.0407	0.7423	0.9001	0.033*	
C11	0.8852 (2)	0.67582 (12)	0.89898 (17)	0.0276 (5)	
H11	0.9065	0.6727	0.9720	0.033*	
C12	0.7739 (2)	0.63893 (11)	0.83531 (16)	0.0229 (5)	
H12	0.7199	0.6106	0.8654	0.027*	
C13	0.8664 (2)	0.61490 (11)	0.20000 (16)	0.0200 (4)	
C14	0.8967 (2)	0.56077 (12)	0.14644 (16)	0.0244 (5)	
H14	0.8345	0.5233	0.1297	0.029*	
C15	1.0187 (2)	0.56155 (13)	0.11728 (17)	0.0275 (5)	
H15	1.0403	0.5238	0.0816	0.033*	
C16	1.1103 (2)	0.61635 (12)	0.13915 (16)	0.0262 (5)	
C17	1.0790 (2)	0.66962 (12)	0.19497 (17)	0.0255 (5)	
H17	1.1417	0.7069	0.2127	0.031*	
C18	0.9572 (2)	0.66934 (11)	0.22540 (17)	0.0222 (4)	
H18	0.9367	0.7062	0.2633	0.027*	
C19	1.2364 (3)	0.61845 (15)	0.0991 (2)	0.0380 (6)	
H19A	1.2087	0.6383	0.0297	0.046*	
H19B	1.2714	0.5725	0.0961	0.046*	
H19C	1.3109	0.6459	0.1456	0.046*	
C20	0.7003 (2)	0.45865 (10)	0.24260 (16)	0.0190 (4)	
C21	0.5841 (2)	0.47540 (11)	0.15902 (16)	0.0211 (4)	
H21	0.5468	0.5199	0.1518	0.025*	
C22	0.5235 (2)	0.42619 (11)	0.08651 (17)	0.0228 (5)	
H22	0.4445	0.4377	0.0293	0.027*	
C23	0.5747 (2)	0.36063 (11)	0.09503 (18)	0.0251 (5)	
C24	0.6912 (2)	0.34575 (11)	0.17858 (19)	0.0277 (5)	
H24	0.7287	0.3013	0.1858	0.033*	
C25	0.7547 (2)	0.39399 (11)	0.25185 (18)	0.0233 (5)	
H25	0.8350	0.3827	0.3081	0.028*	
C26	0.5040 (3)	0.30853 (13)	0.0151 (2)	0.0374 (6)	
H26A	0.5641	0.2684	0.0240	0.045*	
H26B	0.4892	0.3272	−0.0543	0.045*	
H26C	0.4129	0.2961	0.0238	0.045*	
H1N	0.313 (2)	0.4242 (11)	0.4505 (17)	0.010 (5)*	

### Atomic displacement parameters (Å^2^)

**Table:**

	*U*^11^	*U*^22^	*U*^33^	*U*^12^	*U*^13^	*U*^23^
Cu1	0.00918 (16)	0.01796 (18)	0.02017 (18)	0.00287 (13)	−0.00105 (13)	−0.00585 (14)
P1	0.0135 (2)	0.0205 (3)	0.0162 (2)	−0.00016 (19)	0.0050 (2)	0.0009 (2)
S1	0.0133 (2)	0.0297 (3)	0.0214 (3)	0.0016 (2)	0.0055 (2)	0.0021 (2)
S2	0.0159 (2)	0.0296 (3)	0.0201 (3)	−0.0048 (2)	0.0055 (2)	−0.0039 (2)
O1	0.0156 (7)	0.0284 (8)	0.0194 (7)	0.0006 (6)	0.0062 (6)	0.0053 (6)
O2	0.0194 (7)	0.0201 (7)	0.0179 (7)	0.0027 (6)	0.0034 (6)	−0.0002 (6)
N1	0.0111 (8)	0.0157 (9)	0.0154 (8)	0.0015 (6)	0.0033 (7)	0.0002 (7)
N2	0.0107 (8)	0.0172 (8)	0.0166 (8)	0.0004 (6)	0.0023 (6)	−0.0003 (7)
C1	0.0119 (9)	0.0186 (10)	0.0186 (10)	0.0001 (7)	−0.0003 (8)	−0.0010 (8)
C2	0.0095 (9)	0.0168 (10)	0.0256 (11)	0.0004 (7)	0.0018 (8)	−0.0012 (8)
C3	0.0131 (9)	0.0191 (10)	0.0188 (10)	0.0015 (7)	0.0058 (8)	0.0009 (8)
C4	0.0140 (9)	0.0177 (10)	0.0305 (12)	0.0017 (8)	0.0031 (9)	−0.0067 (9)
C5	0.0127 (9)	0.0196 (10)	0.0178 (10)	−0.0006 (7)	0.0050 (8)	−0.0028 (8)
C6	0.0147 (10)	0.0168 (10)	0.0297 (11)	0.0043 (8)	0.0032 (9)	−0.0003 (8)
C7	0.0136 (9)	0.0188 (10)	0.0169 (10)	0.0016 (7)	0.0012 (8)	−0.0044 (8)
C8	0.0181 (10)	0.0223 (11)	0.0176 (10)	0.0001 (8)	0.0039 (8)	−0.0036 (8)
C9	0.0175 (10)	0.0226 (11)	0.0289 (12)	−0.0023 (8)	0.0050 (9)	−0.0048 (9)
C10	0.0191 (11)	0.0296 (12)	0.0270 (12)	−0.0016 (9)	−0.0030 (9)	−0.0123 (10)
C11	0.0260 (12)	0.0355 (13)	0.0166 (10)	0.0042 (10)	−0.0004 (9)	−0.0054 (9)
C12	0.0219 (11)	0.0277 (12)	0.0183 (10)	0.0014 (9)	0.0051 (9)	0.0000 (9)
C13	0.0154 (10)	0.0290 (12)	0.0162 (10)	0.0004 (8)	0.0059 (8)	0.0057 (8)
C14	0.0240 (11)	0.0312 (12)	0.0194 (11)	−0.0051 (9)	0.0085 (9)	−0.0012 (9)
C15	0.0248 (11)	0.0410 (14)	0.0185 (11)	0.0018 (10)	0.0094 (9)	−0.0002 (10)
C16	0.0170 (10)	0.0449 (14)	0.0154 (10)	0.0006 (9)	0.0030 (8)	0.0102 (10)
C17	0.0203 (11)	0.0318 (12)	0.0221 (11)	−0.0050 (9)	0.0031 (9)	0.0102 (9)
C18	0.0223 (11)	0.0228 (11)	0.0210 (11)	0.0010 (8)	0.0058 (9)	0.0053 (8)
C19	0.0198 (11)	0.0663 (19)	0.0288 (13)	−0.0008 (12)	0.0086 (10)	0.0091 (12)
C20	0.0202 (10)	0.0223 (11)	0.0172 (10)	−0.0033 (8)	0.0094 (8)	−0.0001 (8)
C21	0.0237 (11)	0.0220 (11)	0.0184 (10)	−0.0011 (8)	0.0077 (9)	0.0030 (8)
C22	0.0236 (11)	0.0260 (12)	0.0204 (10)	−0.0077 (9)	0.0093 (9)	0.0008 (9)
C23	0.0280 (12)	0.0274 (12)	0.0268 (12)	−0.0104 (9)	0.0184 (10)	−0.0044 (9)
C24	0.0295 (12)	0.0208 (11)	0.0379 (13)	−0.0010 (9)	0.0178 (11)	0.0008 (10)
C25	0.0217 (10)	0.0225 (11)	0.0277 (11)	0.0013 (8)	0.0104 (9)	0.0039 (9)
C26	0.0385 (14)	0.0340 (14)	0.0445 (16)	−0.0150 (11)	0.0198 (13)	−0.0120 (12)

### Geometric parameters (Å, °)

**Table:**

Cu1—N2	1.9899 (16)	C9—C10	1.384 (3)
Cu1—N2^i^	1.9900 (16)	C9—H9	0.9500
Cu1—N1^i^	2.0074 (16)	C10—C11	1.379 (3)
Cu1—N1	2.0074 (16)	C10—H10	0.9500
Cu1—S1	3.0090 (7)	C11—C12	1.389 (3)
P1—O2	1.6272 (15)	C11—H11	0.9500
P1—O1	1.6283 (15)	C12—H12	0.9500
P1—S2	1.9489 (8)	C13—C14	1.379 (3)
P1—S1	1.9577 (8)	C13—C18	1.383 (3)
O1—C13	1.407 (2)	C14—C15	1.385 (3)
O2—C20	1.388 (2)	C14—H14	0.9500
N1—C5^i^	1.475 (2)	C15—C16	1.391 (3)
N1—C1	1.479 (2)	C15—H15	0.9500
N1—H1N	0.81 (2)	C16—C17	1.388 (3)
N2—C3	1.274 (3)	C16—C19	1.510 (3)
N2—C2	1.477 (2)	C17—C18	1.392 (3)
C1—C2	1.516 (3)	C17—H17	0.9500
C1—H1A	0.9900	C18—H18	0.9500
C1—H1B	0.9900	C19—H19A	0.9800
C2—H2A	0.9900	C19—H19B	0.9800
C2—H2B	0.9900	C19—H19C	0.9800
C3—C6	1.502 (3)	C20—C25	1.383 (3)
C3—C4	1.503 (3)	C20—C21	1.393 (3)
C4—C5	1.526 (3)	C21—C22	1.387 (3)
C4—H4A	0.9900	C21—H21	0.9500
C4—H4B	0.9900	C22—C23	1.388 (3)
C5—N1^i^	1.475 (2)	C22—H22	0.9500
C5—C7	1.521 (3)	C23—C24	1.386 (3)
C5—H5	1.0000	C23—C26	1.508 (3)
C6—H6A	0.9800	C24—C25	1.386 (3)
C6—H6B	0.9800	C24—H24	0.9500
C6—H6C	0.9800	C25—H25	0.9500
C7—C12	1.383 (3)	C26—H26A	0.9800
C7—C8	1.401 (3)	C26—H26B	0.9800
C8—C9	1.387 (3)	C26—H26C	0.9800
C8—H8	0.9500		
			
N2—Cu1—N2^i^	179.999 (1)	C9—C8—C7	120.6 (2)
N2—Cu1—N1^i^	95.10 (7)	C9—C8—H8	119.7
N2^i^—Cu1—N1^i^	84.90 (7)	C7—C8—H8	119.7
N2—Cu1—N1	84.91 (7)	C10—C9—C8	120.1 (2)
N2^i^—Cu1—N1	95.10 (7)	C10—C9—H9	120.0
N1^i^—Cu1—N1	180.00 (9)	C8—C9—H9	120.0
N2—Cu1—S1	79.57 (5)	C11—C10—C9	119.8 (2)
N2^i^—Cu1—S1	100.43 (5)	C11—C10—H10	120.1
N1^i^—Cu1—S1	83.94 (5)	C9—C10—H10	120.1
N1—Cu1—S1	96.06 (5)	C10—C11—C12	120.2 (2)
O2—P1—O1	102.03 (8)	C10—C11—H11	119.9
O2—P1—S2	105.09 (6)	C12—C11—H11	119.9
O1—P1—S2	111.92 (6)	C7—C12—C11	120.9 (2)
O2—P1—S1	111.82 (6)	C7—C12—H12	119.5
O1—P1—S1	105.98 (6)	C11—C12—H12	119.5
S2—P1—S1	118.81 (4)	C14—C13—C18	120.6 (2)
P1—S1—Cu1	106.29 (3)	C14—C13—O1	119.69 (19)
C13—O1—P1	120.20 (12)	C18—C13—O1	119.67 (19)
C20—O2—P1	127.06 (13)	C13—C14—C15	119.4 (2)
C5^i^—N1—C1	113.27 (16)	C13—C14—H14	120.3
C5^i^—N1—Cu1	115.14 (12)	C15—C14—H14	120.3
C1—N1—Cu1	106.15 (12)	C14—C15—C16	121.5 (2)
C5^i^—N1—H1N	110.0 (15)	C14—C15—H15	119.3
C1—N1—H1N	108.3 (15)	C16—C15—H15	119.3
Cu1—N1—H1N	103.3 (15)	C17—C16—C15	118.1 (2)
C3—N2—C2	120.32 (16)	C17—C16—C19	121.5 (2)
C3—N2—Cu1	127.88 (14)	C15—C16—C19	120.4 (2)
C2—N2—Cu1	111.48 (12)	C16—C17—C18	121.1 (2)
N1—C1—C2	108.76 (16)	C16—C17—H17	119.5
N1—C1—H1A	109.9	C18—C17—H17	119.5
C2—C1—H1A	109.9	C13—C18—C17	119.4 (2)
N1—C1—H1B	109.9	C13—C18—H18	120.3
C2—C1—H1B	109.9	C17—C18—H18	120.3
H1A—C1—H1B	108.3	C16—C19—H19A	109.5
N2—C2—C1	108.72 (15)	C16—C19—H19B	109.5
N2—C2—H2A	109.9	H19A—C19—H19B	109.5
C1—C2—H2A	109.9	C16—C19—H19C	109.5
N2—C2—H2B	109.9	H19A—C19—H19C	109.5
C1—C2—H2B	109.9	H19B—C19—H19C	109.5
H2A—C2—H2B	108.3	C25—C20—O2	115.45 (19)
N2—C3—C6	123.65 (18)	C25—C20—C21	120.1 (2)
N2—C3—C4	121.68 (18)	O2—C20—C21	124.44 (19)
C6—C3—C4	114.57 (17)	C22—C21—C20	119.0 (2)
C3—C4—C5	119.40 (17)	C22—C21—H21	120.5
C3—C4—H4A	107.5	C20—C21—H21	120.5
C5—C4—H4A	107.5	C21—C22—C23	122.0 (2)
C3—C4—H4B	107.5	C21—C22—H22	119.0
C5—C4—H4B	107.5	C23—C22—H22	119.0
H4A—C4—H4B	107.0	C24—C23—C22	117.6 (2)
N1^i^—C5—C7	112.89 (16)	C24—C23—C26	122.3 (2)
N1^i^—C5—C4	110.59 (16)	C22—C23—C26	120.1 (2)
C7—C5—C4	109.37 (16)	C25—C24—C23	121.7 (2)
N1^i^—C5—H5	107.9	C25—C24—H24	119.1
C7—C5—H5	107.9	C23—C24—H24	119.1
C4—C5—H5	107.9	C20—C25—C24	119.6 (2)
C3—C6—H6A	109.5	C20—C25—H25	120.2
C3—C6—H6B	109.5	C24—C25—H25	120.2
H6A—C6—H6B	109.5	C23—C26—H26A	109.5
C3—C6—H6C	109.5	C23—C26—H26B	109.5
H6A—C6—H6C	109.5	H26A—C26—H26B	109.5
H6B—C6—H6C	109.5	C23—C26—H26C	109.5
C12—C7—C8	118.42 (19)	H26A—C26—H26C	109.5
C12—C7—C5	120.90 (19)	H26B—C26—H26C	109.5
C8—C7—C5	120.67 (18)		
			
O2—P1—S1—Cu1	56.88 (6)	C3—C4—C5—C7	−172.28 (18)
O1—P1—S1—Cu1	167.26 (6)	N1^i^—C5—C7—C12	−120.0 (2)
S2—P1—S1—Cu1	−65.82 (4)	C4—C5—C7—C12	116.4 (2)
N2—Cu1—S1—P1	152.82 (5)	N1^i^—C5—C7—C8	60.8 (2)
N2^i^—Cu1—S1—P1	−27.17 (5)	C4—C5—C7—C8	−62.8 (2)
N1^i^—Cu1—S1—P1	56.49 (5)	C12—C7—C8—C9	−0.7 (3)
N1—Cu1—S1—P1	−123.51 (5)	C5—C7—C8—C9	178.54 (18)
O2—P1—O1—C13	−59.27 (16)	C7—C8—C9—C10	−0.1 (3)
S2—P1—O1—C13	52.61 (16)	C8—C9—C10—C11	0.6 (3)
S1—P1—O1—C13	−176.42 (13)	C9—C10—C11—C12	−0.4 (3)
O1—P1—O2—C20	−62.66 (17)	C8—C7—C12—C11	0.8 (3)
S2—P1—O2—C20	−179.58 (14)	C5—C7—C12—C11	−178.35 (19)
S1—P1—O2—C20	50.21 (17)	C10—C11—C12—C7	−0.3 (3)
N2—Cu1—N1—C5^i^	152.28 (14)	P1—O1—C13—C14	88.0 (2)
N2^i^—Cu1—N1—C5^i^	−27.72 (14)	P1—O1—C13—C18	−94.8 (2)
S1—Cu1—N1—C5^i^	73.36 (13)	C18—C13—C14—C15	−0.6 (3)
N2—Cu1—N1—C1	26.10 (13)	O1—C13—C14—C15	176.68 (19)
N2^i^—Cu1—N1—C1	−153.90 (13)	C13—C14—C15—C16	−1.2 (3)
S1—Cu1—N1—C1	−52.82 (12)	C14—C15—C16—C17	2.5 (3)
N1^i^—Cu1—N2—C3	5.34 (18)	C14—C15—C16—C19	−175.1 (2)
N1—Cu1—N2—C3	−174.66 (18)	C15—C16—C17—C18	−2.0 (3)
S1—Cu1—N2—C3	−77.53 (17)	C19—C16—C17—C18	175.5 (2)
N1^i^—Cu1—N2—C2	178.79 (13)	C14—C13—C18—C17	1.0 (3)
N1—Cu1—N2—C2	−1.21 (13)	O1—C13—C18—C17	−176.24 (18)
S1—Cu1—N2—C2	95.92 (13)	C16—C17—C18—C13	0.3 (3)
C5^i^—N1—C1—C2	−173.11 (16)	P1—O2—C20—C25	−176.35 (14)
Cu1—N1—C1—C2	−45.81 (17)	P1—O2—C20—C21	6.4 (3)
C3—N2—C2—C1	150.36 (18)	C25—C20—C21—C22	0.7 (3)
Cu1—N2—C2—C1	−23.66 (19)	O2—C20—C21—C22	177.87 (18)
N1—C1—C2—N2	46.1 (2)	C20—C21—C22—C23	0.4 (3)
C2—N2—C3—C6	−2.5 (3)	C21—C22—C23—C24	−1.0 (3)
Cu1—N2—C3—C6	170.41 (15)	C21—C22—C23—C26	179.1 (2)
C2—N2—C3—C4	−178.72 (18)	C22—C23—C24—C25	0.5 (3)
Cu1—N2—C3—C4	−5.8 (3)	C26—C23—C24—C25	−179.6 (2)
N2—C3—C4—C5	−27.8 (3)	O2—C20—C25—C24	−178.60 (19)
C6—C3—C4—C5	155.67 (19)	C21—C20—C25—C24	−1.2 (3)
C3—C4—C5—N1^i^	62.8 (2)	C23—C24—C25—C20	0.6 (3)

Symmetry codes: (i) −*x*+1, −*y*+1, −*z*+1.

### Hydrogen-bond geometry (Å, °)

**Table:**

*D*—H···*A*	*D*—H	H···*A*	*D*···*A*	*D*—H···*A*
N1—H1N···S2^i^	0.81 (2)	2.73 (2)	3.4868 (19)	157
C6—H6C···S2^ii^	0.98	2.80	3.755 (2)	164

Symmetry codes: (i) −*x*+1, −*y*+1, −*z*+1; (ii) *x*−1, *y*, *z*.
